# Lanthanum(III)hydroxide Nanoparticles and Polyethyleneimine-Functionalized Graphene Quantum Dot Nanocomposites in Photosensitive Silicon Heterojunctions

**DOI:** 10.1021/acsami.4c02102

**Published:** 2024-04-18

**Authors:** Aslıhan Anter, Elif Orhan, Murat Ulusoy, Barış Polat, Mustafa Yıldız, Arun Kumar, Antonio Di Bartolomeo, Enver Faella, Maurizio Passacantando, Jinshun Bi

**Affiliations:** † Department of Physics, Gazi University, Ankara 06500, Türkiye; ‡ Industrial Engineering, Ankara Medipol University, Ankara 06050, Türkiye; § Department of Chemistry, Çanakkale Onsekiz Mart University, Çanakkale 17100, Türkiye; ∥ Department of Physics “E.R. Caianiello”, University of Salerno, Fisciano, Salerno 84084, Italy; ⊥ Department of Physical and Chemical Science, University of L’Aquila, Coppito, L’Aquila 67100, Italy; # Institute of Microelectronics, Chinese Acedemy Science (CAS), Beijing 10010, China

**Keywords:** rare earth elements, lanthanum(III) hydroxide doping, graphene quantum dots, green method, nanocomposite diode, photosensitivity

## Abstract

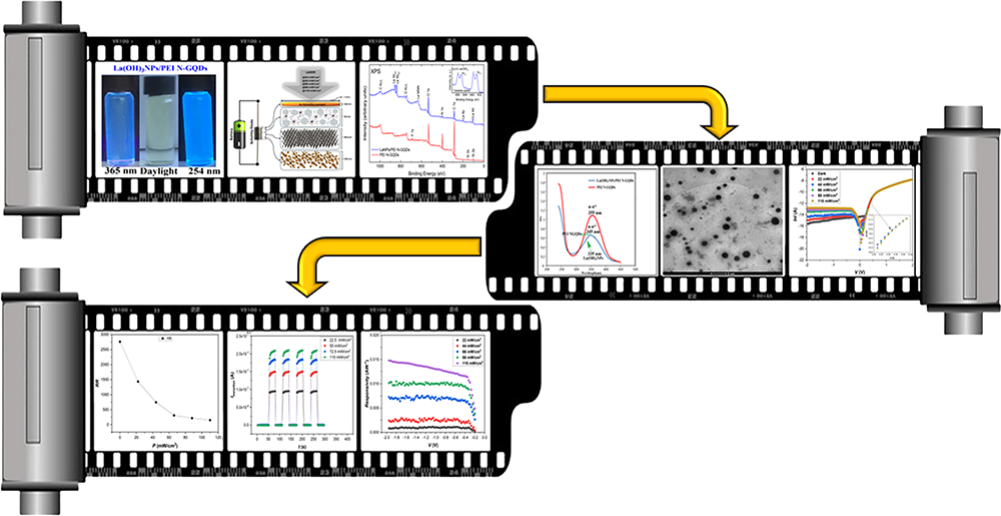

Lanthanides are largely used in optoelectronics as dopants to enhance the physical and optical properties of semiconducting devices. In this study, lanthanum(III)hydroxide nanoparticles (La(OH)_3_NPs) are used as a dopant of polyethylenimine (PEI)-functionalized nitrogen (N)-doped graphene quantum dots (^*PEI-N*^*GQDs*). The *La(OH)*_*3*_*NPs-doped*^*PEI-N*^*GQDs* nanocomposites are prepared from La(NO)_3_ in a single step by a green novel method and are characterized by Fourier-transform infrared spectroscopy (FT-IR), ultraviolet–visible spectroscopy (UV–vis), X-ray photoelectron spectroscopy (XPS), and transmission electron microscopy (TEM). Deposited over an n-type Si wafer, the *La(OH)*_*3*_*NPs-doped*^*PEI-N*^*GQDs* nanocomposites form Schottky diodes. The *I*–*V* characteristics and the photoresponse of the diodes are investigated as a function of the illumination intensity in the range 0–110 mW cm^–2^ and at room temperature. It is found that the rectification ratio and ideality factor of the diode decrease, while the Schottky barrier and series resistance increase with the enhancing illuminations. As a photodetector, the *La(OH)*_*3*_*NPs-doped*^*PEI-N*^*GQDs/n-Si* heterojunction exhibits an appreciable responsivity of 3.9 × 10^–3^ AW^–1^ under 22 mW cm^–2^ at −0.3 V bias and a maximum detectivity of 8.7 × 10^8^ Jones under 22 mW cm^–2^ at −0.5 V. This study introduces the green synthesis and presents the structural, electrical, and optoelectronic properties of *La(OH)*_*3*_*NPs-doped*^*PEI-N*^*GQDs*, demonstrating that these nanocomposites can be promising for optoelectronic applications.

## 1 Introduction

Technological progress largely depends on the development of semiconductor-based devices. The Schottky barrier diode (SBD) is formed by the junction of a metal with a semiconductor and is very useful in various applications like rectifiers, filters, and integrated circuits.^1−4^ The SBD can include a native thin interface between the metal and the semiconductor that converts the MS structure into a metal–interlayer–semiconductor (MIS) structure.^5−8^ The performance, stability, and reliability of the SBD are significantly influenced by this interlayer, which is crucial for the regulation of electrical parameters.^9−12^ Lately, there has been an increasing interest in SBD that includes a carbon nanostructured layer inserted between a metal and semiconductor. Carbon-based nanostructures,^13^ in particular graphene quantum dots (*GQDs*),^14^ have had a profound impact on several fields including optoelectronic devices, biosensors, and energy conversion systems.^15^*GQDs* exhibit remarkable properties, including fluorescence properties, biocompatibility, versatility in synthesis from various organic precursors, low toxicity, abundant functional groups, tunable emission wavelength, photostability, effortless surface modification, and chemical inertness.^16,17^*GQDs* are functionalized in various ways to extend their application in various fields.^18^ The functionalization of *GQDs* is performed through doping with heteroatoms or by forming composites with inorganic materials or polymers and can alter their optical, chemical, and electronic properties.^19−23^ Nanocomposites of *GQDs* functionalized with polyethylenimine *(PEI-GQDs*) have been synthesized and used for optoelectronic applications^9,24^ as well as for cancer theragnostic and nucleic acid delivery.^25−28^ A review of the literature shows interesting and impressive results from Schottky diodes that exploit rare earth elements (REEs)-doped *GQDs-*based materials. REEs are an excellent choice for doping GQDs. They lead to the creation of hybrid materials that combine the favorable properties of GQDs and REEs to enhance their luminescence properties, applicability, and quantum yield, opening the door to a wide range of practical and technological applications.^9^ For instance, Orhan et al. fabricated a Gd-doped PEI-functionalized *N*-doped GQDs (*Gd-doped*^*PEI-N*^*GQDs*) nanocomposite diode with enhanced electrical properties and high photoluminescence (PL) quantum yield (PLQY).^9^ Lanthanum (La)-based materials have been proven to be particularly promising. La is the first REE of the lanthanide series and is arranged in the [Xe]5d^1^6s^2^ configuration with 57 electrons. The luminescence of lanthanides and their compounds has been used in many technological applications, such as color televisions, fluorescent lamps, energy-saving lamps, cameras, and telescope lenses.^29,30^ Lanthanide luminescent lifetimes are typically on the millisecond time scale, exceeding those observed for organic fluorophores, and may be useful for time-gated monitoring applications.^17,31^ Shao et al.^32^ showed that the performance of diamond SBD can be significantly improved by inserting an ultrathin lanthanum hexaboride (LaB_6_) layer followed by a rapid thermal annealing (RTA) treatment. Liu et al.^33^ investigated the electrical and hydrogen sensing properties of a Schottky diode based on a Pd/La-WO_3_/SiC structure, indicating that the presence of La significantly improves the Schottky diode hydrogen sensitivity.

Despite its technological potential, La(OH)_3_ doping of functionalized GQDs has still been poorly explored and requires further investigation.

In this study, *La(OH)*_*3*_*NPs-doped*^*PEI-N*^*GQDs* nanocomposites were prepared by a green method in a single step from the reaction of La(NO_3_)_3_ with ^*PEI-N*^*GQDs* in a water bath at 90 °C, and Fourier-transform infrared spectroscopy (FT-IR), ultraviolet–visible spectroscopy (UV–vis), X-ray photoelectron spectroscopy (XPS), and transmission electron microscopy (TEM). The electrical characteristics and the photodetection of the SBDs obtained by depositing the *La(OH)*_*3*_*NPs-doped*^*PEI-N*^*GQDs* nanocomposite onto n-type Si are investigated as a function of the illumination intensity. It is shown that the *La(OH)*_*3*_*NPs-doped*^*PEI-N*^*GQDs/Si* diodes achieve good rectification and responsivity and are promising for optoelectronic applications.

## 2 Materials and Methods

### 2.1 Materials

All chemicals were obtained from commercial sources and used without further purification. The citric acid (CA), La(NO_3_)_3_·6H_2_O, and polyethylenimine (PEI) (*M*_w_: 1300, 50 wt % in H_2_O) were purchased from Sigma-Aldrich. The synthesized nanocomposite solutions were characterized by using complementary methods. Infrared absorption (IR) spectra were obtained from a PerkinElmer BX II FT (Fourier Transmission)-IR spectrometer on KBr discs. The UV–visible spectra were measured by using a PG Instruments T+80 UV–visible spectrometer. XPS studies of the ^*PEI-N*^*GQDs* and *La(OH)*_*3*_*NPs-doped*^*PEI-N*^*GQDs* nanocomposite materials were performed using the PHI ESCA system equipped with an Mg Kα photon source (*h*ν = 1253.6 eV) and a hemispherical analyzer. Binding energy data were calibrated using the Ag 3d_5/2_ signal peak (368.3 eV) obtained by a small silver dot deposited onto each sample. CTEM analysis was carried out by an FEI Technai G^2^ Spirit BioTwin, 120 kV electron microscope. A specimen was prepared by sonification of the *La(OH)*_*3*_*NPs-doped*^*PEI-N*^*GQDs* nanocomposite solution in DI water. A single droplet of the solution was dropped onto the carbon film-supported copper grid. The specimen was dried and analyzed. We finally examined the photodetection performance of the fabricated diode. A continuum white light source (NKT Photonics – SuperK COMPACT) was used to investigate the sensitivity (*S*), responsivity *R*, and specific detectivity (D*) of the photodetector under illumination in the range 22–110 mW cm^–2^. *La(OH)*_*3*_*NPs-doped*^*PEI-N*^*GQDs* nanocomposites were morphologically characterized by high contrast transmission electron microscopy (CTEM) (FEI Technai G^2^ Spirit BioTwin) at an accelerating voltage of 120 kV.

### 2.2 Synthesis of *PEI-functionalized N-doped GQDs*(^*PEI-N*^*GQDs*) and *La(OH)*_*3*_*NPs-doped*^*PEI-N*^*GQDs* nanocomposites

PEI-functionalized N-doped GQDs were successfully synthesized by a hydrothermal process, which is a green method.^9,34,35^ The CA (1.80 g, 8.57 mmol) and PEI (3.71 g, 2.85 mmol) were dissolved in 50 mL of deionized water in a Teflon container and placed in an autoclave. The autoclave was kept in an oven at 200 °C for 18 h. The suspension products in the autoclave cooled to room temperature were centrifuged at 12000 rpm for 10 min, and nanoparticles were collected. The collected ^*PEI-N*^*GQDs* nanoparticles were washed twice with deionized water and once with ethanol. The obtained ^*PEI-N*^*GQDs* were dried in a vacuum oven and stored in a desiccator. The synthesis of ^*PEI-N*^*GQDs* is also detailed in our previous work.^9,34,35^

**Scheme 1:**
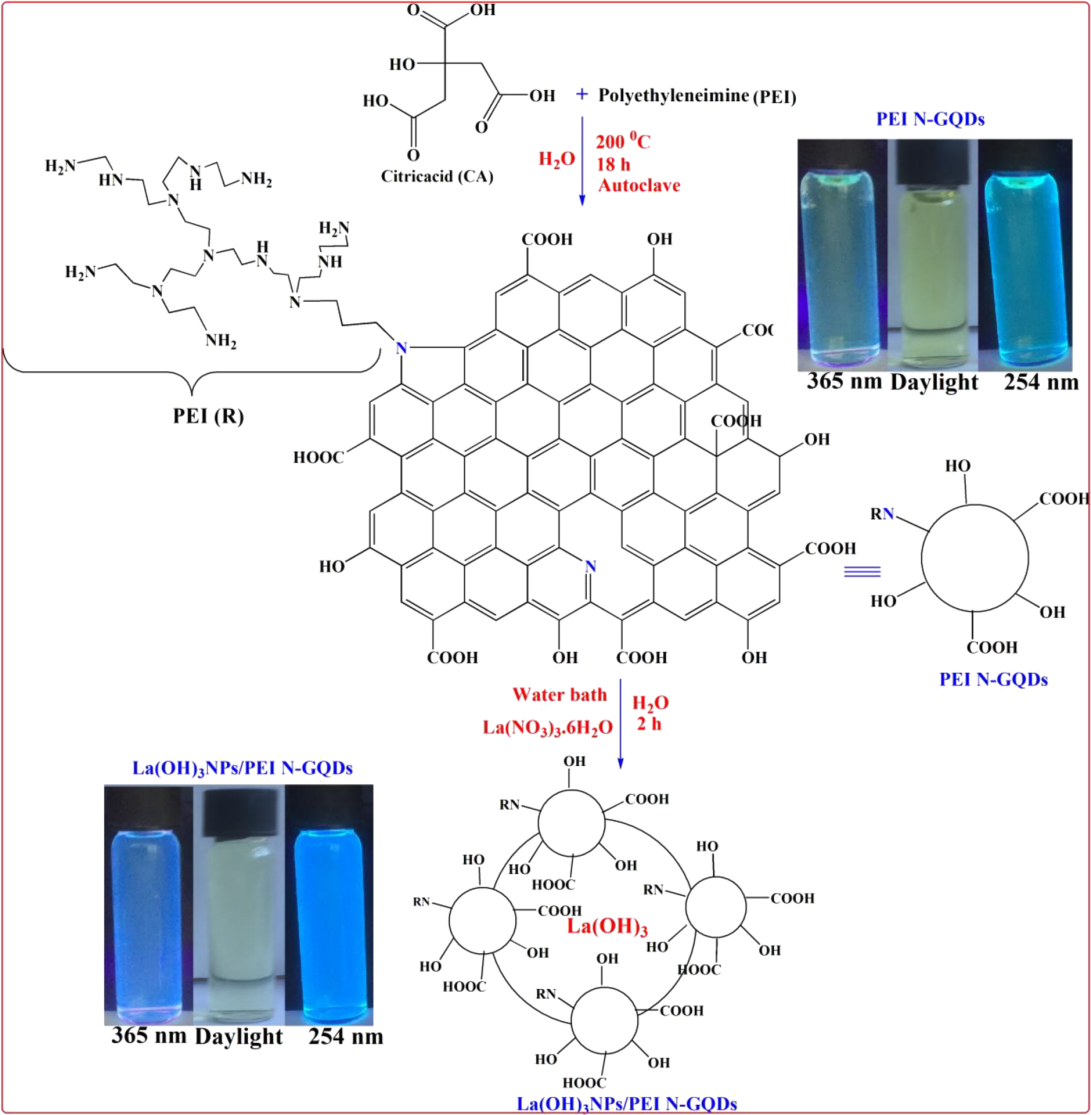
Synthesis of *La(OH)*_*3*_*NPs-doped*^*PEI-N*^*GQDs* Nanocomposites

For the synthesis of La(OH)_3_ nanoparticles and their nanocomposites, a solution of *^PEI-N^GQDs* (1 g) in 100 mL water was added to a 250 mL round-bottom flask. Then, 20 mL of a 0.1 M La(NO_3_)_3_ solution was added into the *^PEI-N^GQDs* mixture. The mixture was heated at 90 °C for 2 h to complete the transformation process of La(NO_3_)_3_ into La(OH)_3_ nanoparticles from lanthanum(III)nitrate and *^PEI-N^GQDs* to form *La(OH)_3_NPs-doped^PEI-N^GQDs* nanocomposites. *La(OH)_3_NPs-doped^PEI-N^GQDs* nanocomposite solutions then were cooled to room temperature, the suspension products were centrifuged at 12000 rpm for 10 min, and the supernatant was collected. *La(OH)_3_NPs-doped^PEI-N^GQDs* nanocomposites were washed once with deionized distilled water and ethanol. The nanocomposites were stored in a desiccator for later use. Interestingly, while La(0) nanometal particles (LaNPs) were expected to be formed in the reaction, La(NO_3_)_3_ was completely converted to La(OH)_3_ by the catalytic effect of *^PEI-N^GQDs* in an aqueous medium. *^PEI-N^GQDs* do not reduce La(III) to La(0) but convert it to La(OH)_3_ via PEI-ammonium hydroxides formed in an aqueous medium. *^PEI-N^GQDs* acted both as catalysts for the formation of La(OH)_3_ and as stabilizers for the nanoparticles formed. In a literature study, it was reported that La(OH)_3_ was synthesized from Li(NO_3_)_3_ in a single step in the presence of hexamethylenetetramine (HMTA) at 95 °C in an autoclave for 8 h.^36^ The synthesis of *La(OH)_3_-doped*^*PEI-N*^*GQDs* nanocomposites is presented in Scheme 1.

### 2.3 Fabrication of the *La(OH)*_*3*_*NPs-doped*^*PEI-N*^*GQDs*/n-Si Diode

**Figure 1:**
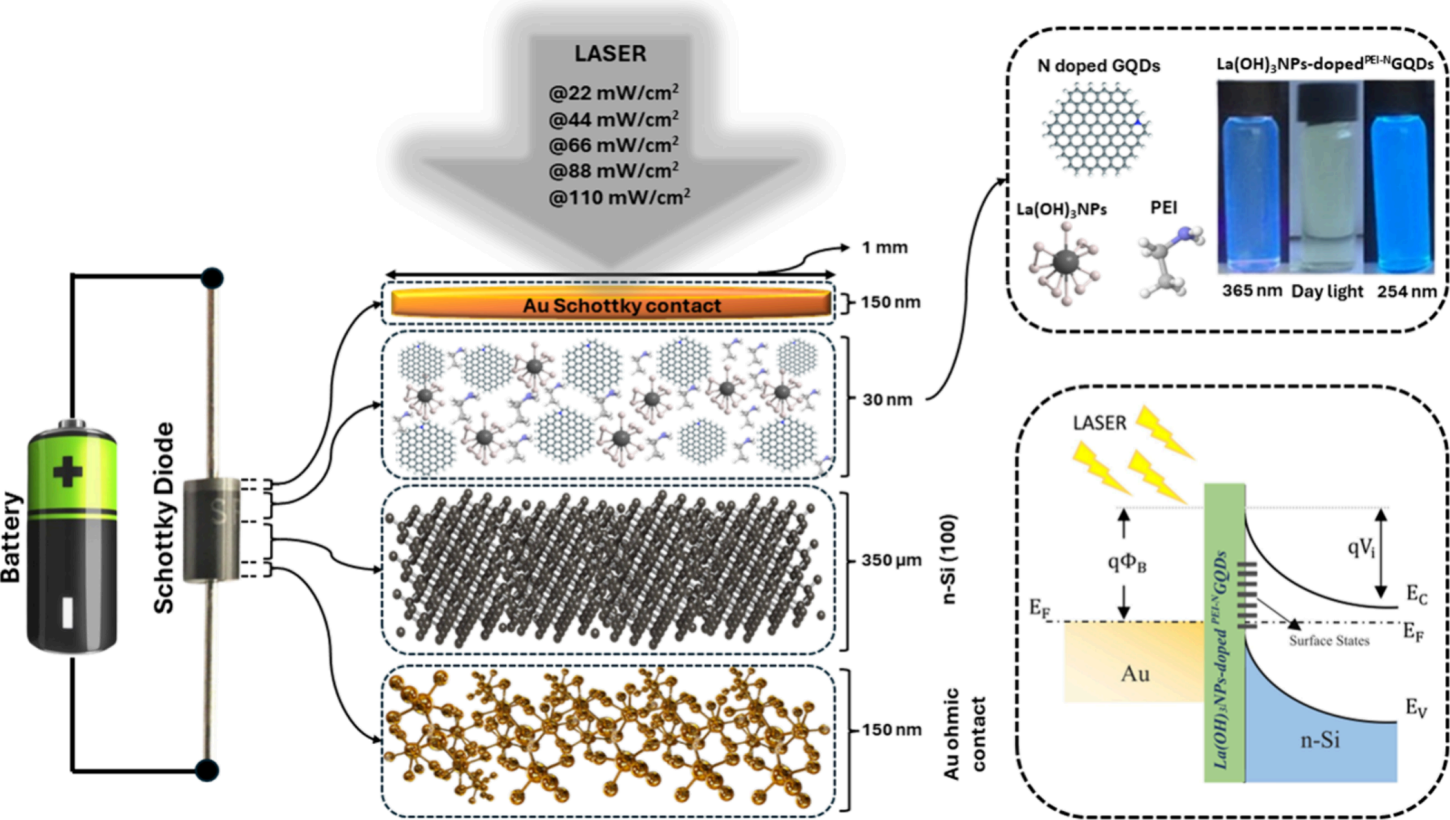
Schematic layout and energy band diagram of the *La(OH)*_*3*_*NPs-doped*^*PEI-N*^*GQDs/n-type Si* heterojunction.

For the fabrication of the diode, n-type Si (1–10 Ωcm, 350 μm thickness) wafers (100) were used. Following cleaning procedures, Au (99.999% pure) with 150 nm thickness was sputtered on the unpolished surface of the n-Si wafer to form an ohmic contact.^34,35^ A spin-coating technique was then used to deposit the resulting *La(OH)*_*3*_*NPs-doped*^*PEI-N*^*GQDs* nanocomposite solution onto the polished surface of the wafer. The spinning speed was 3000 rpm, and spinning time was locked for 30 s. The resulting thin film thickness was 30 nm. Finally, circular Au contacts with a thickness of 150 nm were formed on *La(OH)*_*3*_*NPs-doped*^*PEI-N*^*GQDs* – *n type-*Si by sputtering using a circular metal mask with a diameter of 0.5 mm. Figure 1 shows the *La(OH)*_*3*_*NPs-doped*^*PEI-N*^*GQDs–n-type Si* diode structure. The fabrication of a ^*PEI-N*^*GQDs/n-Si* control device proceeded similarly.

## 3 Results and Discussion

### 3.1 FT-IR, UV–vis, XPS, and TEM Characterizations of ^*PEI-N*^*GQDs* and *La(OH)*_*3*_*NPs-doped*^*PEI-N*^*GQDs*

**Figure 2:**
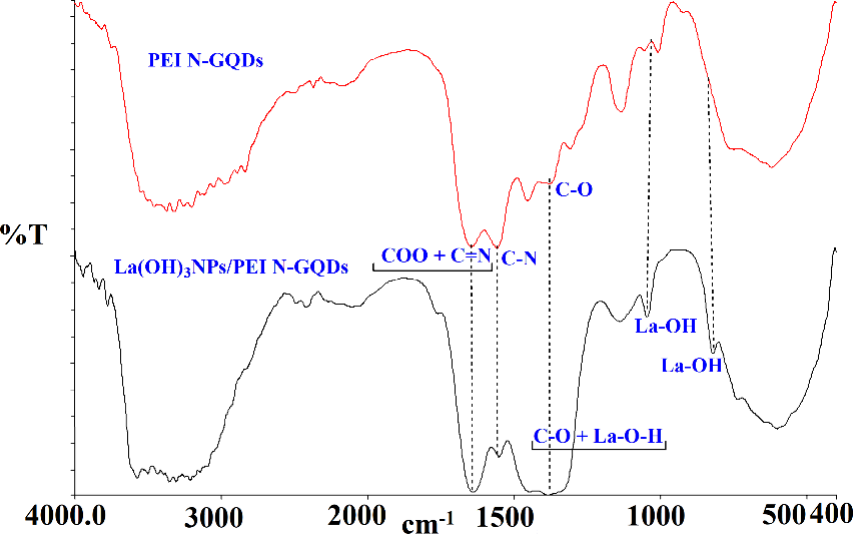
FT-IR spectra of ^*PEI-N*^*GQDs* and *La(OH)*_*3*_*NPs-doped*^*PEI-N*^*GQDs* nanocomposites.

FT-IR spectra of the *La(OH)*_*3*_*NPs-doped*^*PEI-N*^*GQDs* nanocomposites exhibit characteristic changes in the functional group frequencies when compared with the spectrum of the starting materials ^*PEI-N*^*GQDs* (Figure 2). The OH, COOH, NH_2_, NH, C–H, COO+C=N, C=C, C–N, and C–O vibration bands are observed at 3551–3498–3446, 3367, 3320–3200, 3125, 3054, 2967–2839, 1646, 1559, 1454, and 1373 cm^–1^ of starting materials ^*PEI-N*^*GQDs*, respectively. In the *La(OH)*_*3*_*NPs-doped*^*PEI-N*^*GQDs* nanocomposites, the OH, NH_2_, NH, C–H, COO+C=N, C=C, C–N, and C–O vibration bands are observed at 3575–3503–3428, 3353, 3308–3208, 3147, 3046, 2949–2839, 1641, 1551, 1447, and 1385 cm^–1^, respectively. The COO and C=N absorptions overlapped in the FT-IR spectra of all materials. Compared to the starting compound ^*PEI-N*^*GQDs*, in metal nanocomposites, OH, COOH, NH_2_, and NH absorptions shifted to a higher frequency, while C–H, COO+C=N, and C–N absorptions shifted to a lower frequency. The C–O vibration was observed at a higher frequency in *La(OH)*_*3*_*NPs-doped*^*PEI-N*^*GQDs* nanocomposites than in ^*PEI-N*^*GQDs*. The C–O vibration was observed at a higher frequency in *La(OH)*_*3*_*NPs-doped*^*PEI-N*^*GQDs* than in ^*PEI-N*^*GQDs*. Here, both the La–OH bond and the C–O bond were vibrated together at a high frequency. Interestingly, the frequency of carboxyl + imine absorption bands observed at 1646 cm^–1^ in ^*PEI-N*^*GQDs* shifted to the lower frequency of 1641 cm^–1^ in *La(OH)*_*3*_*NPs-doped*^*PEI-N*^*GQDs* nanocomposites. More interestingly, new peaks at 1043 and 840 cm^–1^ were observed in *La(OH)*_*3*_*NPs-doped*^*PEI-N*^*GQDs* nanocomposites, which were not observed in ^*PEI-N*^*GQDs*. This new peak is evidence of the formation of the La–O bond.^37−39^ These data show that lanthanum(III)’ nitrate is converted to La(OH)_3_.

**Figure 3:**
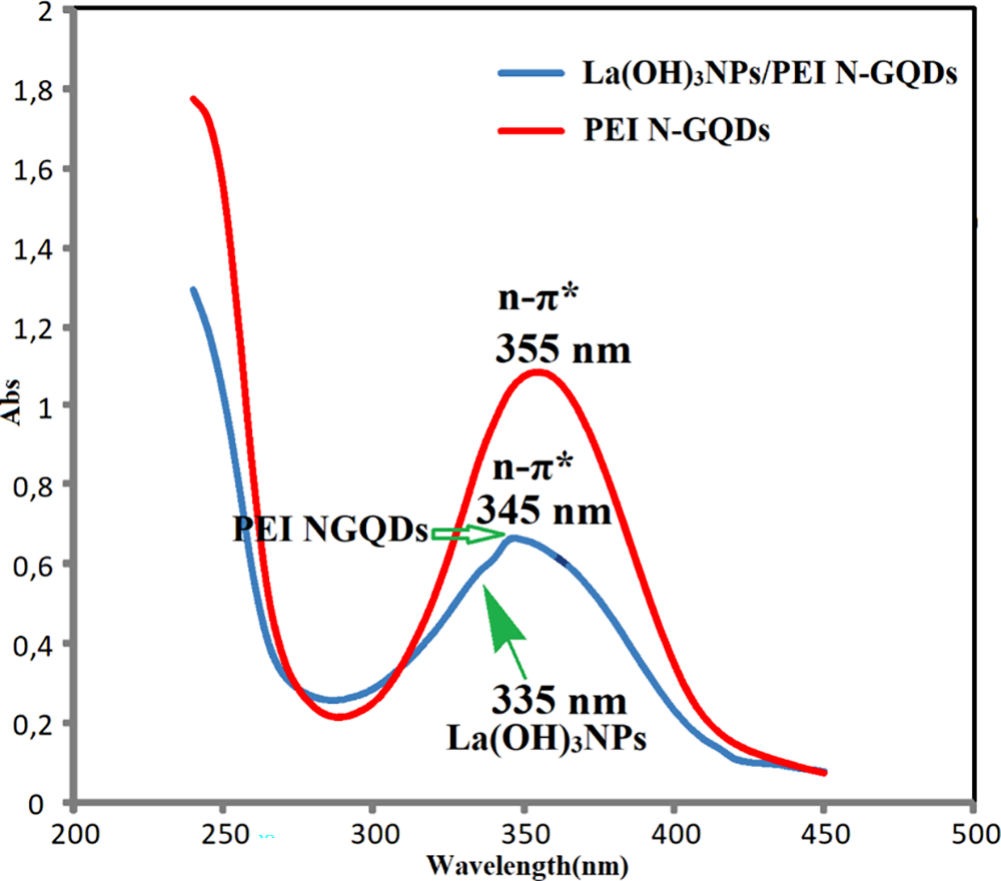
UV–vis spectra of ^*PEI-N*^*GQDs* (red curve) and *La(OH)*_*3*_*NPs-doped*^*PEI-N*^*GQDs* (light blue curve) nanocomposites.

The absorption spectra of suspensions of ^*PEI-N*^*GQDs* and *La(OH)*_*3*_*NPs-doped*^*PEI-N*^*GQDs* nanocomposites in water are shown in Figure 3. One band assigned to n-π* transitions of C=O and C=N is observed at 355 nm in the UV–vis spectrum of *La(OH)*_*3*_*NPs doped*^*PEI-N*^*GQDs* nanocomposites and at 345 nm in the UV–vis spectrum of ^*PEI-N*^*GQDs* nanocomposites. The absorption edge of pure La(OH)_3_ was observed at approximately 250 nm.^40^ After the reaction of La(NO_3_)_3_ with ^*PEI-N*^*GQDs*, the absorption of ^*PEI-N*^*GQDs* was observed to shift to blue (345 nm) in the nanocomposite, indicating the successful formation of *La(OH)*_*3*_*NPs doped*^*PEI-N*^*GQDs* nanocomposites. Also in the nanocomposite, the shoulder at 335 nm was attributed to La(OH)_3_ nanoparticles. The band gap values of pure La(OH)_3_,^40^^*PEI-N*^*GQDs*, and *La(OH)*_*3*_*NPs-doped*^*PEI-N*^*GQDs* nanocomposites are 5.20, 3.10, and 2.90 eV, respectively. The band gap was found to be smaller in the *La(OH)*_*3*_*NPs-doped*^*PEI-N*^*GQDs* nanocomposites, indicating that the nanocomposite is a better conductor.

**Figure 4:**
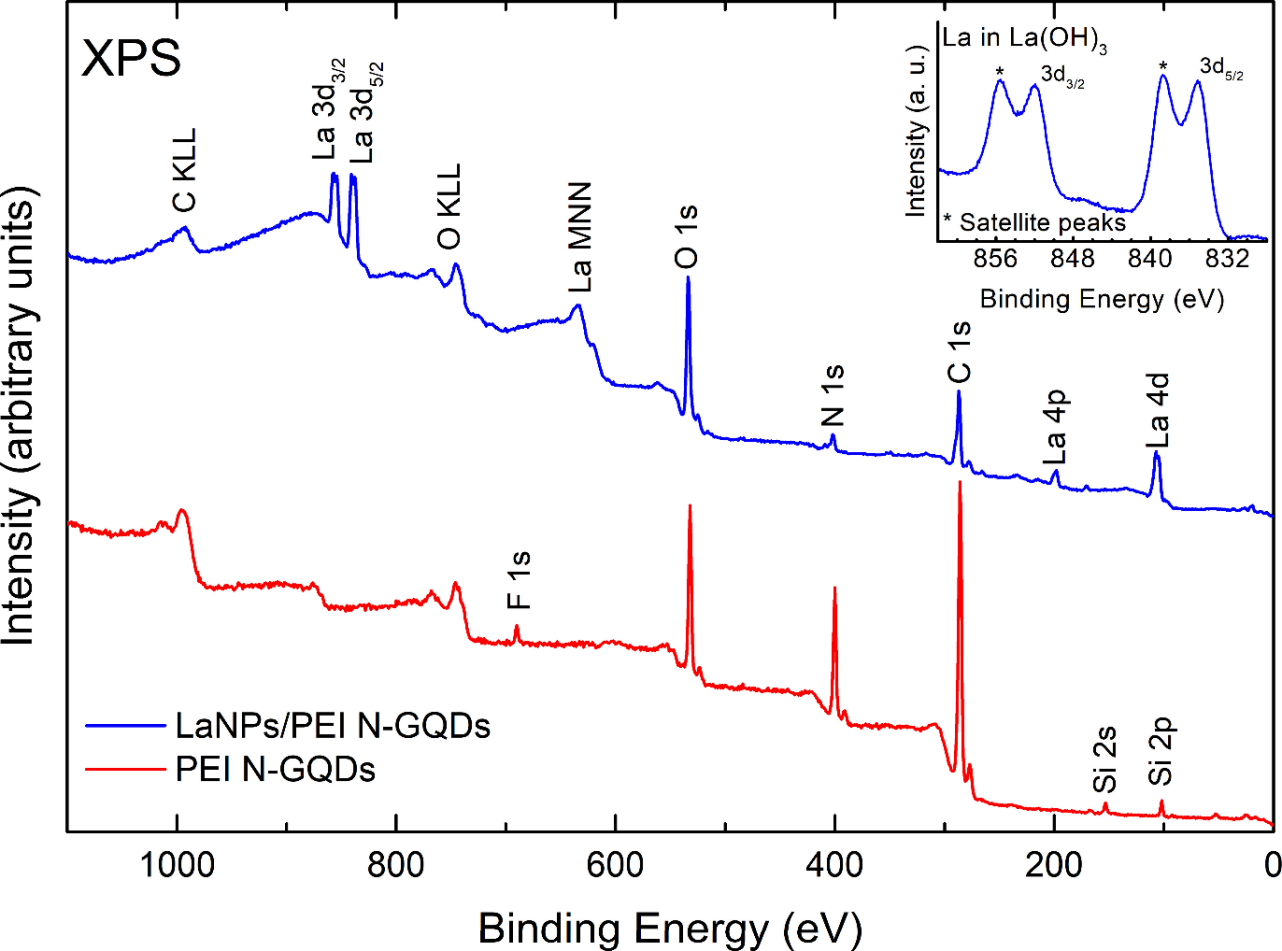
XPS survey spectra of *La(OH)*_*3*_*NPs-doped*^*PEI-N*^*GQDs* (blue curve) and ^*PEI-N*^*GQDs* (red curve). The inset shows the magnification of the La 3d region.

XPS was also used to characterize the synthesized nanomaterials. Figure 4 shows the survey spectra of *La(OH)*_*3*_*NPs-doped*^*PEI-N*^*GQDs* (black curve) and ^*PEI-N*^*GQDs* (red curve). No contaminant species are detectable within the sensitivity of the technique.

From the La 3d, C 1s, O 1s, and N 1s peaks, it is possible to obtain the exact composition of the nanocomposite by calculating the atomic concentration of the individual species using respective sensitivity factors. For the *La(OH)*_*3*_*NPs-doped PEI-NGQDs* sample, the atomic percentage concentrations of 68%, 24%, 7%, and 1% were obtained for C, O, N, and La, respectively. The inset of Figure 4 displays a magnification of the La 3d region confirming the formation of La(OH)_3_.^41^

In conclusion, from FTIR, UV–vis, and XPS data, it can be said that La is La^3+^ in the *La(OH)*_*3*_*NPs-doped*^*PEI-N*^*GQDs* nanocomposite.

**Figure 5:**
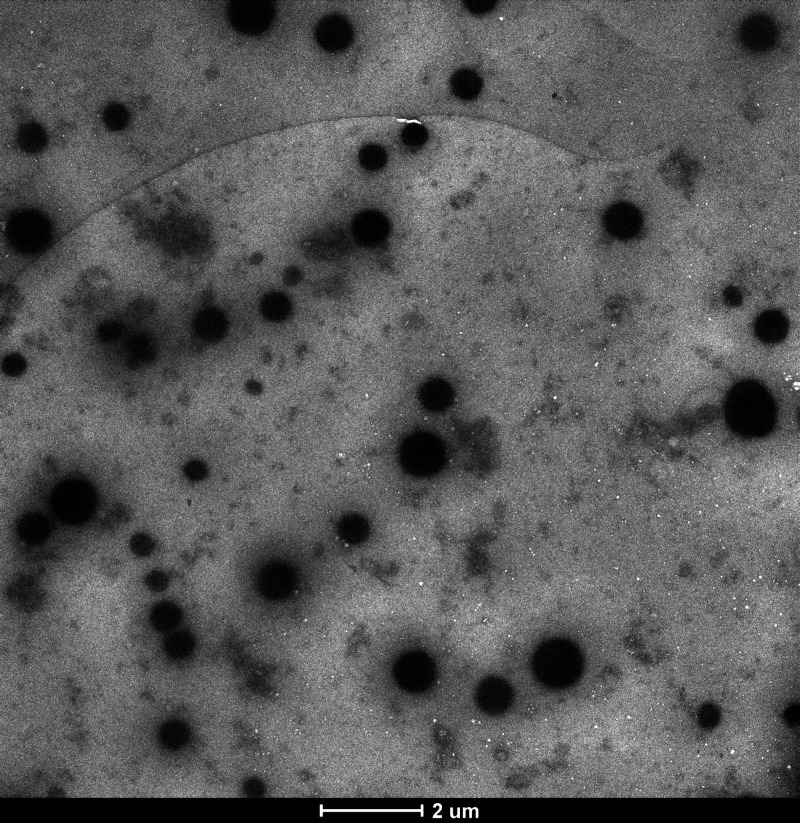
TEM image of the *La(OH)*_*3*_*NPs-doped*^*PEI-N*^*GQDs* nanocomposite.

TEM image of La(OH)_3_NPs doped PEI-NGQDs nanocomposites is shown in Figure 5. TEM analysis shows that La(OH)_3_NPs in the nanocomposite have a spherical structure, with an average size of 6–20 nm. PEI-NGQDs appear as distorted spherical elongated shapes with smaller sizes among La(OH)_3_NPs. It can also be said that the particles form random and partial aggregates.

### 3.2 Electrical Characterization

Current–voltage (*I*–*V*) measurements in the dark and under light were performed to determine the electrical properties of the *La(OH)*_*3*_*NPs-doped*^*PEI-N*^*GQDs***/***n-Si* Schottky diode. Thermionic emission (TE) theory^2,42−44^ and Cheung’s method^45^ were used to estimate the parameters of the diode. The relationship between the TE current and the applied voltage is expressed as follows:
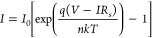
1where *I*_0_, *n*, *V*, *k*, *q*, *IR*_*s*_, and *T* are the reverse saturation current at zero bias, ideal factor, applied bias voltage, Boltzmann constant, electronic charge, voltage drop across series resistance (*R*_*s*_), and temperature in Kelvin, respectively. The reverse saturation current at zero bias, *I*_0_, can be obtained from the ln*I* intercept of the straight-line fitting the linear part of the ln *I*–*V* curve at low forward bias, where the effect of the series resistance *R*_*s*_ is negligible. Then, *I*_0_ can be used to extract the Schottky barrier height φ_*B*_ from the equation:
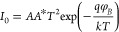
2where A* is the Richardson constant, that is 112 A cm^–2^ K^–2^ for n-type Si at room temperature, and *A* is the area of the heterojunction.

Similarly, the ideality factor *n* can be obtained from the slope of the semilog *I*–*V* curve at low forward bias as
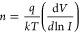
3

The Cheung method offers an alternative to find *n*, *R*_*s*_*,* and φ_*B*_ using the higher forward bias region of the *I*–*V* characteristic, where the effect of *R*_*s*_ is not negligible and ln *I*–*V* shows a downward curvature.

The Cheung method utilizes the following two current- and voltage-dependent functions:
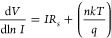
4
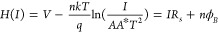
5

The calculations of the Cheung functions, henceforth referred to as Cheung-1 (d*V*/d ln*I*) and Cheung-2 (*H*(*I*)), are described in detail elsewhere.^46−48^

**Figure 6:**
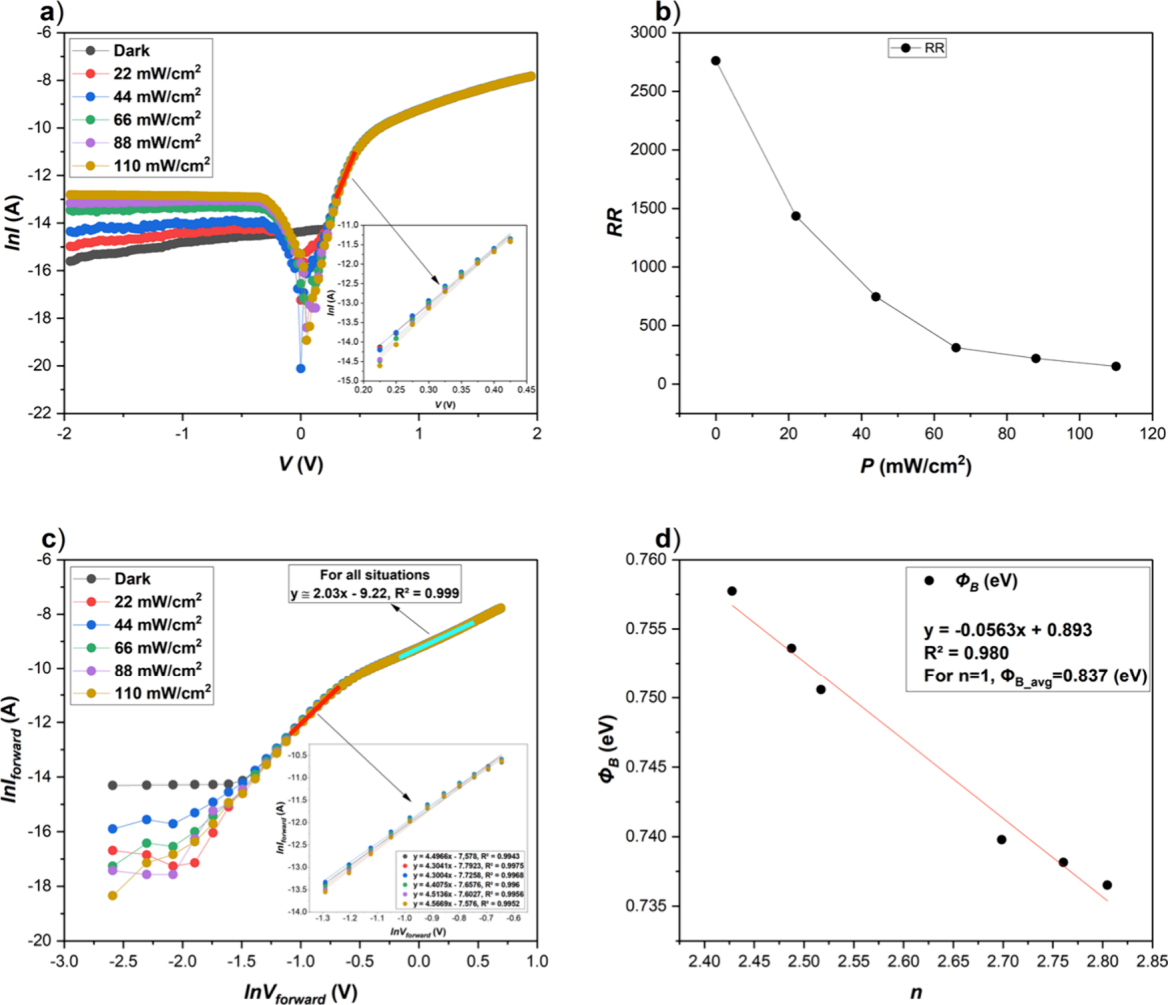
(a) ln *I* vs *V*, (b) *RR* vs light intensity, (c) ln *I*_*forward*_ vs ln *V*_*forward*_, and (d) ϕ_*B*_ vs *n* from TE theory for the *La(OH)*_*3*_*NPs-doped*^*PEI-N*^*GQDs* nanocomposite diode under different illumination intensities.

Figure 6a–d reports the plots of ln *I vs V*, rectification ratio *RR* vs light intensity, ln *I*_forward_ vs ln *V*_*forward*_, and φ_*B*_ vs *n* for the *La(OH)*_*3*_*NPs-doped*^*PEI-N*^*GQDs/n-Si* diode in the dark and under different illumination intensities (*P*) from 22 to 110 mW cm^–2^. Figure 6a shows rectifying ln *I*–*V* characteristics with the current increasing for an increasing illumination intensity. Remarkably, the current reaches a good saturation at reverse biases both in the dark and under illumination. The inset of Figure 6a shows the linear region used to extract the diode parameters using the TE method. The increased current under illumination is due to electron–hole pair photogeneration. Under reverse bias, a photocurrent growing with the enhanced illumination intensity is observed because the electron–hole pairs, photogenerated in the extended depletion region of the *La(OH)*_*3*_*NPs-doped*^*PEI-N*^*GQDs/n-Si* heterojunction, are efficiently separated by the strong electric field (internal + external electric fields). Conversely, in forward bias, the weakened electric field (internal – external electric fields) and the reduced depletion region result in a current under illumination that is indistinguishable from the dark current. Hence, the rectification ratio, RR(*V*) = *I*(*V*)/*I*(−*V*), decreases with increasing illumination, as shown in Figure 6b.

Figure 6c shows the ln *I*_*forward*_ vs ln *V*_*forward*_ plot under different illumination intensities. This plot gives essential clues about the dominant current conduction mechanisms in the forward bias. The linear parts of the double-logarithmic current–voltage curve correspond to power law relationships (*I*_*forward*_ ∝ *V*_*forward*_^*m*^), with the exponent ″*m*″ indicating different conduction mechanisms. At high biases, for *V* > 0.5 V, the value of *m* approaches 2, indicating a space-charge-limited current trend.^49^

The basic parameters of the *La(OH)*_*3*_*NPs-doped*^*PEI-N*^*GQDs***/***n-Si* diode obtained from TE theory and Cheung-1 or Cheung-2 functions are shown in Table 1 as a function of the illumination intensity. Notably, Table 1 shows that the ideality factor *n* decreases while the Schottky barrier ϕ_*B*_ increases with the growing light intensity. Figure 6d shows that there is an anticorrelation between *n* and φ_*B*_. This behavior can be attributed to spatial inhomogeneities of the Schottky barrier, which may result from lattice defects and/or surface impurities.^50^ At low illumination, electrons cross the barrier mainly at the minima of the barrier height, thus resulting in lower average φ_*B*_ and higher *n*.^51−53^

**Table 1:** Basic Diode Parameters of the *La(OH)*_*3*_*NPs-doped*^*PEI-N*^*GQDs* Nanocomposite Diode

**TE**						**Cheung-1 (d***V***/dln***I***)**		**Cheung-2 (***H***(***I***))**	
*P***(mW.cm**^**–2**^**)**	*R*_*s*_**(kΩ)**	**RR**	*I*_**0**_**(nA)**	***n***	φ_*B*_**(eV)**	***n***	*R*_*s***1**_**( kΩ)**	φ_*B*_**(eV)**	*R*_*s***2**_**(kΩ)**
**0**	4.8	2760	34.6	2.80	0.737	3.31	6.9	0.698	4.3
**22**	4.8	1435	32.5	2.76	0.738	3.46	6.9	0.691	4.1
**44**	4.7	746	30.5	2.70	0.740	3.62	6.9	0.686	4.0
**66**	4.8	311	20.1	2.52	0.751	3.57	6.9	0.688	4.0
**88**	4.8	219	17.9	2.49	0.754	3.48	6.9	0.691	4.0
**110**	4.8	152	15.2	2.43	0.758	3.46	6.9	0.692	4.0

**Figure 7:**
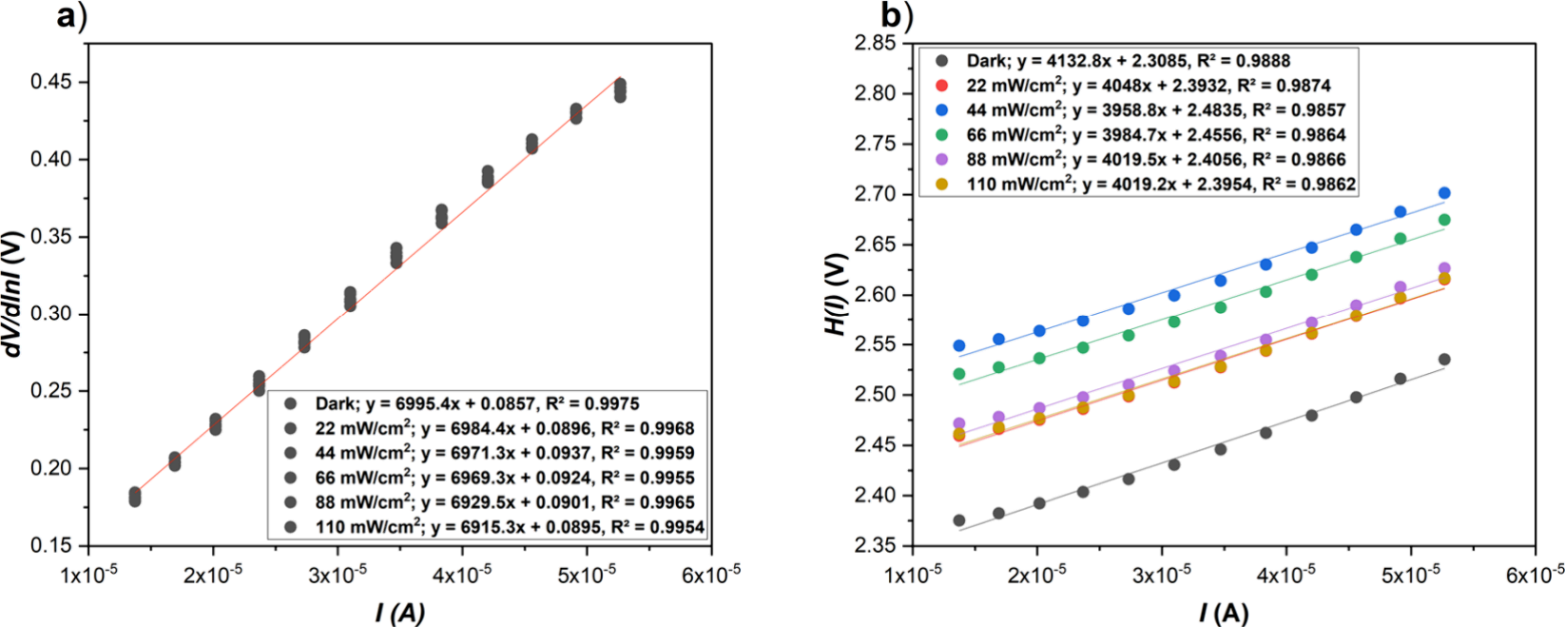
(a) Cheung-1 and (b) Cheung-2 plots of the *La(OH)*_*3*_*NPs-doped*^*PEI-N*^*GQDs* nanocomposite diode at different intensities of illuminations.

The *n* and ϕ_*B*_ values obtained from the TE method are 2.80 and 0.737 eV in the dark. Cheung-1 and Cheung-2 plots of the *La(OH)*_*3*_*NPs-doped*^*PEI-N*^*GQDs* nanocomposite diode are shown in Figures 7a,b. The series resistances *R*_*s*_ of the structure are obtained from the slopes of these plots, and in the dark, results are 2.1 kΩ (Cheung-1) and 1.7 kΩ (Cheung-2), respectively. Using eq 5, the ideality factor *n =* 3.31 is extracted from the intercept point of the *y*-axis of the plot. Substituting this *n* value into eq 5, and ϕ_*B*_ = 0.698 eV is obtained. These values are consistent with TE ones.

Berktaş et al. investigated the *I*–*V*^34^ and C/(G/ω)-V^35^ properties of the *^PEI-N^GQDs* nanocomposite-based diode across a frequency range of 1 kHz to 2 MHz and voltage range of −3 to +7 V. Orhan et al. examined the electrical properties and photoluminescence quantum yield (PLQY) of *^PEI-N^GQDs* doped with the rare earth element Gd.^9^ The PLQYs of GdNPs ^*PEI-N*^*GQDs* (35.96%) were compared with those of the Gd-free ^*PEI-N*^*GQDs* sample (7.60%), revealing a 470% increase in quantum efficiency for the Gd-doped sample. Additionally, it has been noted that the Gd-free ^*PEI-N*^*GQDs* sample exhibits a good rectification ratio (RR: 2.8 × 10^4^, ± 5 V), whereas, after Gd doping, the diode demonstrates ohmic behavior (RR: 14, ± 5 V). Furthermore, the Gd-doped ^*PEI-N*^*GQDs* structure displayed no negative capacitance (NC) behavior across any frequency range,^34^ while NC behavior at low frequencies was observed in the Gd-free ^*PEI-N*^*GQDs* sample.^35^

A comparison of diode parameters for ^*PEI-N*^*GQDs*,^34^*GdNPs-doped*^*PEI-N*^*GQDs*,^9^ and *La(OH)_3_NPs-doped*^*PEI-N*^*GQDs*-based diodes in dark conditions at 300 K is presented in Table 2. Berktaş et al.^34^ reported a rectification ratio (RR) of 2.8 × 10^4^ for the undoped ^*PEI-N*^*GQDs* diode at ±5 V. The findings indicate that the RR of the undoped ^*PEI-N*^*GQDs* diode is 10-fold higher than that of the *La(OH)_3_NPs-doped*^*PEI-N*^*GQDs* diode (RR = 2.8 × 10^3^ at ±2 V). Berktaş et al.^34^ observed an ohmic response with a low RR (14 at ±5 V) for a Gd-doped ^*PEI-N*^*GQDs* diode. Comparing the undoped structure with the lanthanum doped diode, it was observed that the lanthanum doped sample had a lower *n* value approaching the ideal diode value and no significant change (2%) in barrier height for both structures.

**Table 2:** Comparison of Diode Parameters of ^*PEI-N*^*GQDs, GdNPs-*^*PEI-N*^*GQDs,* and *La(OH)*_*3*_*NPs-*^*PEI-N*^*GQDs*-Based Diodes

	**diodes**											
	^*PEI-N*^*GQDs*(34)				*GdNPs*^*PEI-N*^*GQDs*(9)				*La(OH)*_*3*_*NPs-*^*PEI-N*^*GQDs* in the present study			
**methods**	***n***	ϕ_*B*_**(eV)**	**RR**	*R*_*s*_**(kΩ)**	***n***	ϕ_*B*_**(eV)**	**RR**	*R*_*s*_**(kΩ)**	***n***	ϕ_*B*_**(eV)**	**RR**	*R*_*s*_**(kΩ)**
TE	3.71	0.76	2.8 × 10^4^		4.9	0.61	14	3.85	2.8	0.74	2.8 × 10^3^	4.8
Cheung-1 (d*V*/d*l*n I)	9.2	-	-	143	11.6	-	-	2.63	3.31	-	-	6.9
Cheung-2 (H(I))	-	0.66	-	110		0.58		2.35	-	0.70	-	4.3

Surface states (*N*_*ss*_) and their distribution play an important role in the current transport mechanism. These states, which can have different origins and can depend on the illumination intensity, are studied using the method presented by Card and Rhoderick^54^ and obtained from the relation
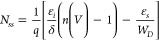
6where *q* is the charge of an electron, ε_s_ and ε_i_ are the dielectric permittivity of the semiconductor and interlayer, respectively, *W_D_* is the depletion layer width, *n*(*V*)is the voltage-dependent ideality factor, and δ is the interfacial layer thickness. The energy levels of the surface states (*E*_*ss*_) are calculated relative to the edge of the conduction band (*E*_*c*_) for n-type Si and are given by
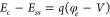
7where φ_*e*_ is the effective barrier height. This is provided by
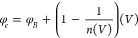
8

**Figure 8:**
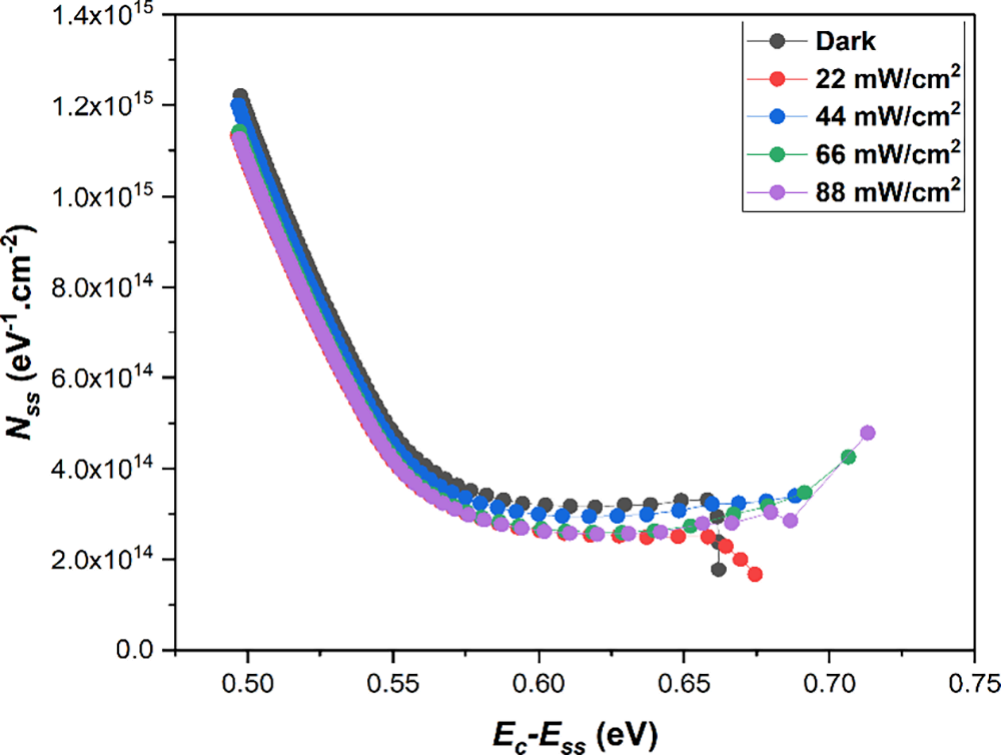
*N*_*ss*_ vs *E*_*c*_–*E*_*ss*_ plots of the *La(OH)*_*3*_*NPs NPs-doped*^*PEI-N*^*GQDs* nanocomposite diode under different illumination intensities.

Figure 8 shows the plots of *N*_*ss*_ as a function of *E*_*c*_ – *E*_*ss*_ obtained from the above equations. The surface states exhibit a decrease that is nearly exponential as the energy difference *E*_*c*_ – *E*_*ss*_ increases. However, with the growing illumination, the concentrations begin to decrease. The illumination that generates electron–hole pairs at the interface also leads to a decrease in the surface states, consistently with the observed decreasing ideality factor.^55−57^

### 3.3 Photodetector’s Figures of Merit

**Figure 9:**
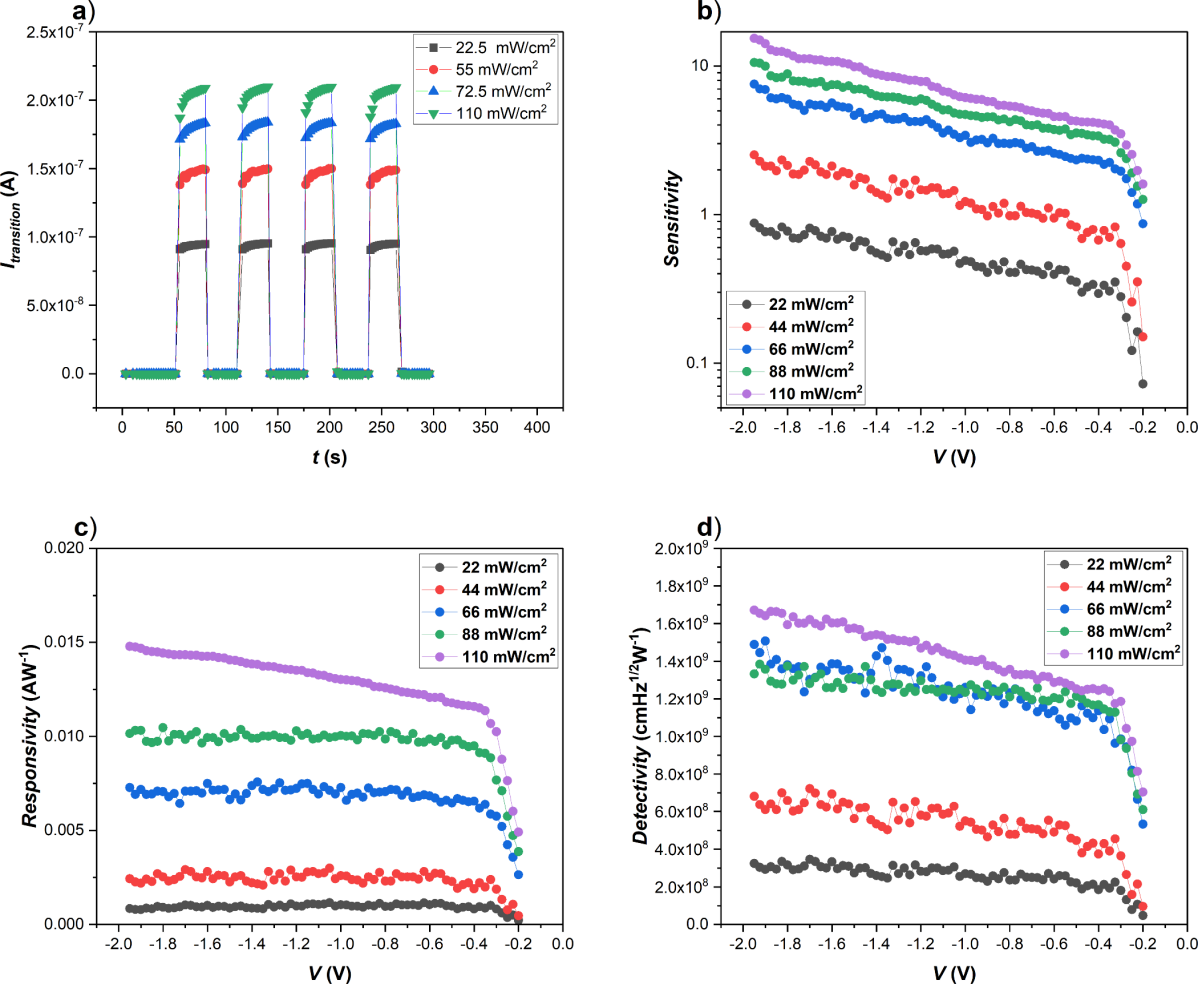
(a) *I*_transient_ vs *t*, (b) sensitivity vs *V*, (c) responsivity vs *V*, and (d) detectivity vs *V* for the *La(OH)*_*3*_*NPs-doped*^*PEI-N*^*GQDs* nanocomposite diode under different illumination intensities.

First, the transient photocurrent (*I*_transient_) characteristics were studied as a function of the increasing light intensity with 30 s long light pulses at zero bias (Figure 9a). The *I*_transient_ increases rapidly with each time the surface is illuminated. As the illumination intensity increases, *I*_transient_ shows stronger time dependence that can be attributed to the distribution of *N*_*ss*_ and their impact on photogeneration.^58−61^

**Figure 10:**
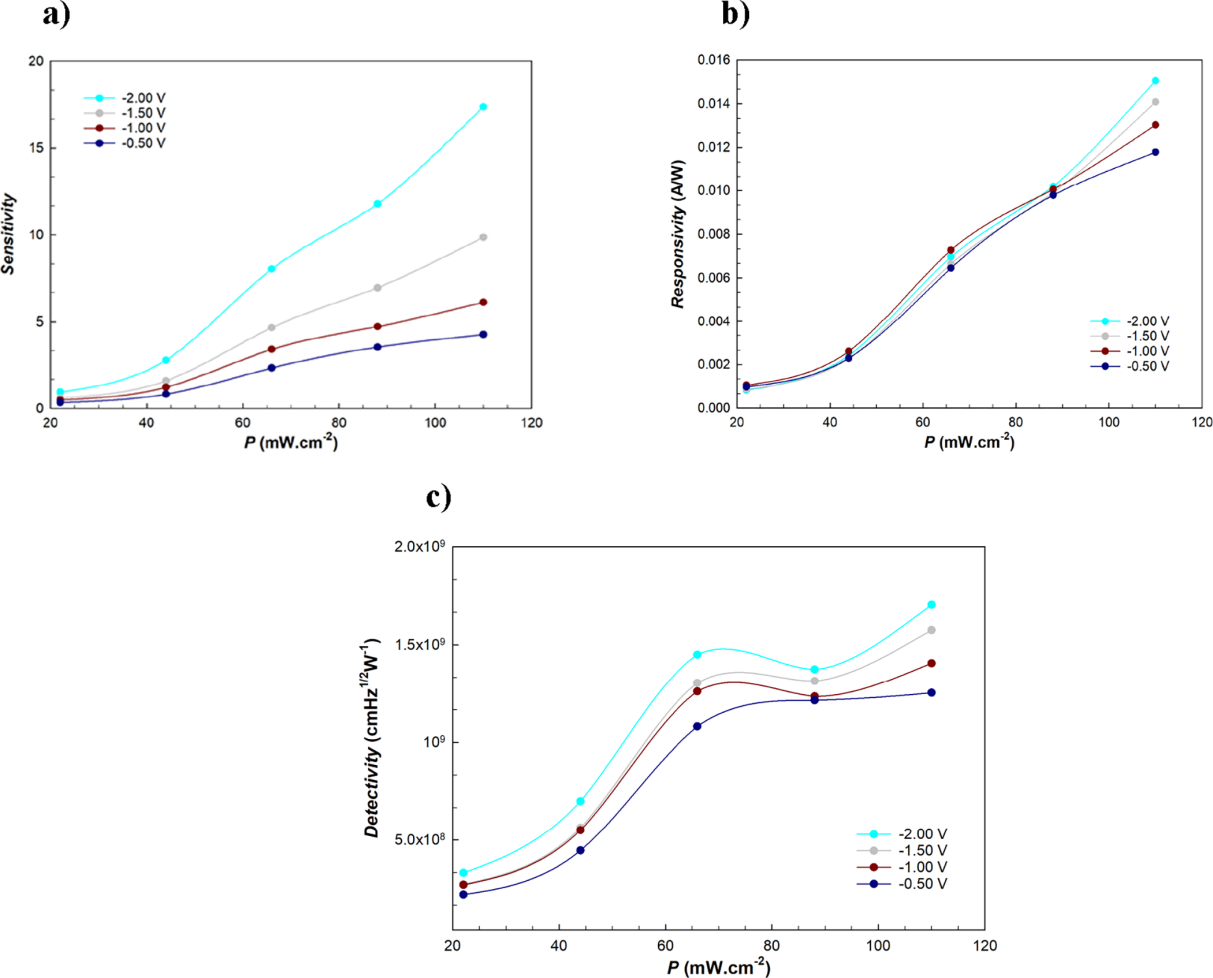
(a) *R vs P* plots, (b) *S* vs *P* plots, and (c) *D* vs *P* plots at certain reverse voltages for *La(OH)*_*3*_*NPs-doped*^*PEI-N*^*GQDs* nanocomposite diode under different illumination intensities.

The ratio of photocurrent to dark current, *S* = (*I*_*light*_*– I*_*dark*_*)/I*_*dark*_, is defined as the sensitivity (*S*) of an optoelectronic device. The *S* curves of the diode are shown in Figure 9b from −2 to 0 V bias under different illumination intensities (*P*). *S* increases as the illumination intensity increases at a given reverse bias. The *La(OH)*_*3*_*NPs-doped*^*PEI-N*^*GQDs* nanocomposite diode shows an increasing sensitivity from 0.94 to 17.37 under 22 and 110 mW cm^–2^ at −2 V, respectively. The *S* versus *P* plots are given for certain reverse voltages in Figure 10a. As can be seen in Figure 10a, the fabricated *La(OH)*_*3*_*NPs-doped*^*PEI-N*^*GQDs* nanocomposite diode exhibits photosensitivity, and the value of photosensitivity increases with illumination intensity.

The responsivity is defined as the ratio of the photocurrent density (*J*_ph_ =*J*_*light*_– *J*_*dark*_) to the incident light intensity and is a measure of the ability of a photodiode to convert incident light into an electrical current:
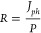
9

The variation of *R* as a function of *P* in reverse bias is shown in Figure 9c. Like *S*, *R* increases for the growing illumination intensity and the reverse bias. The *La(OH)*_*3*_*NPs-doped*^*PEI-N*^*GQDs* nanocomposite diode shows an increasing responsivity from 0.8 to 7.7 mAW^–1^ under 22 and 88 mW cm^–2^ illuminations at −0.3 V, respectively. The *R* versus *P* plots are given for certain reverse voltages in Figure 10b. *R* increases with the illumination intensity. The variation of *R* with the growing reverse bias is due to the enhancing separation of photogenerated electron–hole pairs in the widening depletion layer of the nanocomposite heterojunction.^55−57^

Specific detectivity (D*) is an essential parameter for optoelectronic devices, describing the smallest detectable signal, and can be estimated byeq 10^59−63^
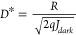
10where *J_dark_* is the current density measured in the dark and D* is in Jones units (Jones = cmHz^0.5^W^1–^). D* is shown in Figure 9d from −2 to 0 V bias at different illuminations. D* increases from 1.8 × 10^8^ to 9.8 × 10^8^ Jones under 22 and 88 mW cm^–2^ illuminations at −0.3 V, respectively. The *D* versus *P* plots are given for certain reverse voltages in Figure 10c. As can be seen in Figure 10c, the diode shows good detectivity, and *D* increases with illumination intensity.

## Conclusions

In this work, we have successfully synthesized *La(OH)*_*3*_*NPs-doped*^*PEI-N*^*GQDs* nanocomposites were prepared by a green method in a single step from the reaction of La(NO_3_)_3_ with ^*PEI-N*^*GQDs* in a water bath at 90 °C. The nanocomposites are characterized by FT-IR, UV–vis, XPS, and TEM analyses. We have fabricated a heterojunction by depositing the *La(OH)*_*3*_*NPs-doped*^*PEI-N*^*GQDs* nanocomposite over n-type Si. We have investigated the electrical behavior and the photoresponse of the heterojunction as a function of the illumination intensity, demonstrating that the device is rectifying and photosensitive. Using the TE and Cheung function method, we have estimated the diode parameters and shown that the Schottky barrier φ_*B*_ increases while the ideality factor *n* decreases for increasing illumination intensity. As a photodetector, the *La(OH)*_*3*_*NPs-doped*^*PEI-N*^*GQDs* nanocomposite diode shows an appreciable responsivity of 3.9 × 10^–3^ AW^–1^ under 22 mW cm^–2^ at −0.3 V and maximum detectivity of 8.7 × 10^8^ Jones under 22 mW cm^–2^ at −0.5 V. This study provides insights into the fabrication and the electrical and photodetection properties of *La(OH)*_*3*_*NPs-doped*^*PEI-N*^*GQDs/n-Si* heterojunction nanocomposite devices.

## Data Availability

All data included in this study are available upon request by contact with the corresponding author.
